# Supplementary material for: Rapid turnover of life-cycle-related genes in the brown algae
Source: Genome Biol. 2019 Feb 14;20:35. doi: 10.1186/s13059-019-1630-6 (PMC6374913; doi:10.1186/s13059-019-1630-6)
Supplement: Supplementary file 3 — Protein alignments, corresponding Gblocks html file and CDS sequences for the lineage conserved gametophyte-biased genes. (ZIP 2109 kb) [file 13059_2019_1630_MOESM3_ESM.zip › Con_GA_alignments/Ec-14_006860.aa_ali.fasta-gb.htm]

Ec-14\_006860.aa.fasta.trans.aa\_ali.fasta


## Gblocks 0.91b Results

Processed file: **Ec-14\_006860.aa.fasta.trans.aa\_ali.fasta**  
Number of sequences: **2**  
Alignment assumed to be: **Protein**  
New number of positions: **616** (selected positions are underlined in blue)

```
                         10        20        30        40        50        60
                 =========+=========+=========+=========+=========+=========+
Ec-14_006860     MDGGLVSNVSGYTVDVERDGFGSAVEPGTEVSVSLVDCVKNQLFEGESDSFPVLKTTLND
MSTRG.54525.1    ------------------------------------------------------------
                                                                             


                         70        80        90       100       110       120
                 =========+=========+=========+=========+=========+=========+
Ec-14_006860     TGISVDEASSAHNSEDQPAAVLFTPGAFSEPPTVELDSLVAGHEGGANVTLALSNPLPAD
MSTRG.54525.1    ------------------------------------------------------------
                                                                             


                        130       140       150       160       170       180
                 =========+=========+=========+=========+=========+=========+
Ec-14_006860     GNIFIEFPSEFSPINCSHVVATGIDGGLEVYVASDAVHTVFEGVSASFPSVKTTLTDVDV
MSTRG.54525.1    ------------------------------------------------------------
                                                                             


                        190       200       210       220       230       240
                 =========+=========+=========+=========+=========+=========+
Ec-14_006860     AIDETSAEFNTPDRPPGVTFTPGAFAGVGPTVVLSTYAAGEVGTANVSFITSNSIPSDGH
MSTRG.54525.1    ------------------------------------------------------------
                                                                             


                        250       260       270       280       290       300
                 =========+=========+=========+=========+=========+=========+
Ec-14_006860     VIITFPSNFTSVDCSVALASGMDGGLVSNVSGYTVDVERDGFGSAVEPGTEVSVSLVDCV
MSTRG.54525.1    ------------------------------------------------------------
                                                                             


                        310       320       330       340       350       360
                 =========+=========+=========+=========+=========+=========+
Ec-14_006860     KNQLFEGESDSFPVLKTTLNDTGISVDEASSAHNSEDQPAAVLFTPGAFSEPPTVELDSL
MSTRG.54525.1    ------------------------------------------------------------
                                                                             


                        370       380       390       400       410       420
                 =========+=========+=========+=========+=========+=========+
Ec-14_006860     VAGHEGGANVTLALSNPLPADGNIFIEFPSEFSPINCSHVVATGIDGGLEVYVASDAVHT
MSTRG.54525.1    ------------------------------------------------------------
                                                                             


                        430       440       450       460       470       480
                 =========+=========+=========+=========+=========+=========+
Ec-14_006860     ASSAHNSEDQPAAVLFTPGAFSEPPTVELDSLVAGHEGGANVTLALSNPLPADGNIFIEF
MSTRG.54525.1    ------------------------------------------------------------
                                                                             


                        490       500       510       520       530       540
                 =========+=========+=========+=========+=========+=========+
Ec-14_006860     PSEFSSINCSHVVATGIDGGLEVYVASDAVHTLFEGVSASFPSVKTTLTDVDVAIDETSA
MSTRG.54525.1    ------------------------------------------------------------
                                                                             


                        550       560       570       580       590       600
                 =========+=========+=========+=========+=========+=========+
Ec-14_006860     EFNTPDRPPGVTFTPGAFAGVGPTVVLSTYAAGEVGTANISFITSNPIPSDGHVIITFPS
MSTRG.54525.1    ------------------------------------------------------------
                                                                             


                        610       620       630       640       650       660
                 =========+=========+=========+=========+=========+=========+
Ec-14_006860     NFTSVDCSVALASGMDGGLVSNVSGYTVDVERDGFGSAVEPGTEVSVSLVDCVKNQLFEG
MSTRG.54525.1    ------------------------------------------------------------
                                                                             


                        670       680       690       700       710       720
                 =========+=========+=========+=========+=========+=========+
Ec-14_006860     ESDSFPVLKTTLNDTGIPVDEASSAQTSEDRPAGVLFTPGAFSEPPTVELDSLVAGHEGG
MSTRG.54525.1    ------------------------------------------------------------
                                                                             


                        730       740       750       760       770       780
                 =========+=========+=========+=========+=========+=========+
Ec-14_006860     ANVTLALSNPLPADGNIFIEFPSEFSPINCSQVVATGIDGGLEVYVASDAVHTLFEGMSA
MSTRG.54525.1    ------------------------------------------------------------
                                                                             


                        790       800       810       820       830       840
                 =========+=========+=========+=========+=========+=========+
Ec-14_006860     SFPSVKTTLTDVDVAIDETSAEFNTPDRRPGVTFTPGAFAGVGPTVVLSTYAAGEVGTAN
MSTRG.54525.1    ------------------------------------------------------------
                                                                             


                        850       860       870       880       890       900
                 =========+=========+=========+=========+=========+=========+
Ec-14_006860     ISFITLNPIPSDGHVIITFPSNFTSVDCSVALASGMDGGLVSNVSGYTVDVERDGFGSAV
MSTRG.54525.1    ------------------------------------------------------------
                                                                             


                        910       920       930       940       950       960
                 =========+=========+=========+=========+=========+=========+
Ec-14_006860     EPGTEVSVSLVDCVKNQLFEGESDSFPVLKTTLNDTGISVDEASSAHNSEDRPAAVLFTP
MSTRG.54525.1    ------------------------------------------------------------
                                                                             


                        970       980       990      1000      1010      1020
                 =========+=========+=========+=========+=========+=========+
Ec-14_006860     GAFSEPPTVELDSLVAGHEGGANVTLALSNPLPADGNIFIEFPSEFSSINCSHVVATGID
MSTRG.54525.1    ------------------------------------------------------------
                                                                             


                       1030      1040      1050      1060      1070      1080
                 =========+=========+=========+=========+=========+=========+
Ec-14_006860     GGLEVYVASDAVHTVRVQRAGDGSVVVGGSSVSLALVDGITNQVFEGVSASFPSVKTTLT
MSTRG.54525.1    ------------------------------------------------------------
                                                                             


                       1090      1100      1110      1120      1130      1140
                 =========+=========+=========+=========+=========+=========+
Ec-14_006860     DVDVAIDETSAEFNTPDRPPGVTFTPGAFAGVGPTVVLSAYAAGEVGTANVSFITSNPIP
MSTRG.54525.1    ------------------------------------------------------------
                                                                             


                       1150      1160      1170      1180      1190      1200
                 =========+=========+=========+=========+=========+=========+
Ec-14_006860     SDGHVIITFPSNFTSVDCSVALASGMDGGLVSNVSGYTVDVERDGFGSAVEPGTEVSVSL
MSTRG.54525.1    ------------------------------------------------------------
                                                                             


                       1210      1220      1230      1240      1250      1260
                 =========+=========+=========+=========+=========+=========+
Ec-14_006860     VDCVKNQLFEGESDSFPVLKTTLNDTGISVDEASSAHNSEDRPAAVLFTPGAFSEPPTVE
MSTRG.54525.1    ------------------------------------------------------------
                                                                             


                       1270      1280      1290      1300      1310      1320
                 =========+=========+=========+=========+=========+=========+
Ec-14_006860     LDSLVAGHEGGANVTLALSNPLPADGNIFIEFPSEFSSINCSHVVATGIDGGLEVYVASD
MSTRG.54525.1    ------------------------------------------------------------
                                                                             


                       1330      1340      1350      1360      1370      1380
                 =========+=========+=========+=========+=========+=========+
Ec-14_006860     AVHTIEFPPSFPSVSPMVADSAELGTVSVNGTFSVSVQRDGLGDIVPTGTSVALILSTVI
MSTRG.54525.1    ------------------------------------------------------------
                                                                             


                       1390      1400      1410      1420      1430      1440
                 =========+=========+=========+=========+=========+=========+
Ec-14_006860     NRGSVGDTGNFSVTTFTDSSMNNRIDVGVAASVVVGKQVLSTAAAFGSSSVAVAAQNKRW
MSTRG.54525.1    ------------------------------------------------------------
                                                                             


                       1450      1460      1470      1480      1490      1500
                 =========+=========+=========+=========+=========+=========+
Ec-14_006860     TFFGHGVALEDKVKWVNSSATSDEDCNWNAEEINIDVFNGSASSAIVTFSESSETTGPLQ
MSTRG.54525.1    ------------------------------------------------------------
                                                                             


                       1510      1520      1530      1540      1550      1560
                 =========+=========+=========+=========+=========+=========+
Ec-14_006860     LCYQFSNGSHPFKLYPAINIDVFELYSVQDAEQGSISLSVVGYAKVLTLSGFGNAEFDEA
MSTRG.54525.1    ------------------------------------------------------------
                                                                             


                       1570      1580      1590      1600      1610      1620
                 =========+=========+=========+=========+=========+=========+
Ec-14_006860     KWLLGSTNCSSARDVAPLASGGDGEVNAASVSSTFQASFEFTEDVFTQAAAGNTSAGATL
MSTRG.54525.1    ------------------------------------------------------------
                                                                             


                       1630      1640      1650      1660      1670      1680
                 =========+=========+=========+=========+=========+=========+
Ec-14_006860     CYRFGSEEFQHYPSISMGILYLNGITSAVGSSSLAVVDVPEILSFSGYGISENASSEDRA
MSTRG.54525.1    ------------------------------------------------------------
                                                                             


                       1690      1700      1710      1720      1730      1740
                 =========+=========+=========+=========+=========+=========+
Ec-14_006860     RWILSGTDCSDNWALISDEVATDGRVEVSSGEATFTFMTSMSGETPSLCYWFQDEPGVLF
MSTRG.54525.1    ------------------------------------------------------------
                                                                             


                       1750      1760      1770      1780      1790      1800
                 =========+=========+=========+=========+=========+=========+
Ec-14_006860     SSVVINVAHLSTLSAPSFGDADVAVVGYPKSWGFSGGHIEDNDFVRWIYNESSDCSESNA
MSTRG.54525.1    ------------------------------------------------------------
                                                                             


                       1810      1820      1830      1840      1850      1860
                 =========+=========+=========+=========+=========+=========+
Ec-14_006860     LPEMEEDGKIISGETTCTFAESVSGRWITPCYRHGSEPWRRYSMPVYVKMVHNMTAVWGS
MSTRG.54525.1    ------------------------------------------------------------
                                                                             


                       1870      1880      1890      1900      1910      1920
                 =========+=========+=========+=========+=========+=========+
Ec-14_006860     DATCVVDQGKKWAFEGDGLQAGDETDLIKFVINDTDCASNSEAPLTGADSDGVAMYLETD
MSTRG.54525.1    ------------------------------------------------------------
                                                                             


                       1930      1940      1950      1960      1970      1980
                 =========+=========+=========+=========+=========+=========+
Ec-14_006860     STATFIVESAAAGYFINLCYKFGNEEFMWYDIRAFAHMVKSVDSLVGGKDIAVVDVEEVL
MSTRG.54525.1    ------------------------------------------------------------
                                                                             


                       1990      2000      2010      2020      2030      2040
                 =========+=========+=========+=========+=========+=========+
Ec-14_006860     VIRANGTSSQDYMRWIVSNETSGTSCNDTAVVLDGPSEGANEIIDMPIFYRGGVFLANFT
MSTRG.54525.1    ------------------------------------------------------------
                                                                             


                       2050      2060      2070      2080      2090      2100
                 =========+=========+=========+=========+=========+=========+
Ec-14_006860     FSASSAGLSPILCYKFADEPWKAYNDVTIDVAMLQEVHANEGSNYVAVVNYSKMWTMAGQ
MSTRG.54525.1    ------------------------------------------------------------
                                                                             


                       2110      2120      2130      2140      2150      2160
                 =========+=========+=========+=========+=========+=========+
Ec-14_006860     FLAAGDQVREEVFWSASTCETASAANLVGDPDGILLVDSTLAPWFAFDSSASGSIVHICY
MSTRG.54525.1    ------------------------------------------------------------
                                                                             


                       2170      2180      2190      2200      2210      2220
                 =========+=========+=========+=========+=========+=========+
Ec-14_006860     KFNDEPFKEYVGFEIDIRMIRAVAVDVGSPRKAIPGVAKTFSFIGDGTATEAGLDQVKWV
MSTRG.54525.1    ------------------------------------------------------------
                                                                             


                       2230      2240      2250      2260      2270      2280
                 =========+=========+=========+=========+=========+=========+
Ec-14_006860     DGDDCLSNGITGDVEDAEGIRSFNLLYFDATGFTAQTIAFPEREIGGPYAMCYSFDEEPY
MSTRG.54525.1    ------------------------------------------------------------
                                                                             


                       2290      2300      2310      2320      2330      2340
                 =========+=========+=========+=========+=========+=========+
Ec-14_006860     QVYVDLVMTVLGISDVTAAEGAANTIVVGAVKRLNFTGGGAVEAGDNAVWVSGGRVDSDC
MSTRG.54525.1    ------------------------------------------------------------
                                                                             


                       2350      2360      2370      2380      2390      2400
                 =========+=========+=========+=========+=========+=========+
Ec-14_006860     LVTAFLSGPESTVDLLNGADFFIPDEDGEGMAGRTWTLCYRFGTESFKIYTSFTLTAVRL
MSTRG.54525.1    ------------------------------------------------------------
                                                                             


                       2410      2420      2430      2440      2450      2460
                 =========+=========+=========+=========+=========+=========+
Ec-14_006860     VDFSAEDGSDDDSVVGYPKTFHPSGFGIAEGDKSKWVMASVSSDEGCESDGVVDEEQVEW
MSTRG.54525.1    ------------------------------------------------------------
                                                                             


                       2470      2480      2490      2500      2510      2520
                 =========+=========+=========+=========+=========+=========+
Ec-14_006860     TMYTPALVTANTSTVDPMTTTSSGSSAVATSSPEEEVLSSTFTFTTPTFSSTRVQLCYKH
MSTRG.54525.1    ------------------------------------------------------------
                                                                             


                       2530      2540      2550      2560      2570      2580
                 =========+=========+=========+=========+=========+=========+
Ec-14_006860     QDEPYHLHAGITLRTRQLLSATIRELGTEQVLTSITNSPQVVAFVAVGGMEGDQYKWVPA
MSTRG.54525.1    ------------------------------------------------------------
                                                                             


                       2590      2600      2610      2620      2630      2640
                 =========+=========+=========+=========+=========+=========+
Ec-14_006860     TESYTSETLLDLCADSVDPAAGSLVGVAAGFYQEASFTFTEPVSDLMLCYAPGSEPFMPY
MSTRG.54525.1    ------------------------------------------------------------
                                                                             


                       2650      2660      2670      2680      2690      2700
                 =========+=========+=========+=========+=========+=========+
Ec-14_006860     PNITMEALAPVIATSNTTHVIAGRSTTVRLIGTFGLTSGDALKLADNADGDCEGNAAGGD
MSTRG.54525.1    ------------------------------------------------------------
                                                                             


                       2710      2720      2730      2740      2750      2760
                 =========+=========+=========+=========+=========+=========+
Ec-14_006860     EAEFAPDTTVLGSSGPALGTCTITLYVSDRTEENRPYKLCYRFGAAGVWELFENVSLEAY
MSTRG.54525.1    ------------------------------------------------------------
                                                                             


                       2770      2780      2790      2800      2810      2820
                 =========+=========+=========+=========+=========+=========+
Ec-14_006860     EVTGVSMDNDGEGSPAAGDALQFSFSGTGIVDGDLAKWVGASTSSDIQCDAATPAFGSAT
MSTRG.54525.1    ------------------------------------------------------------
                                                                             


                       2830      2840      2850      2860      2870      2880
                 =========+=========+=========+=========+=========+=========+
Ec-14_006860     ATVVGGVAEFAFDANVEDWVLCYKHGSDDWRFYAAIVPVSVSSTSSSGTTAISDTQRTKA
MSTRG.54525.1    ------------------------------------------------------------
                                                                             


                       2890      2900      2910      2920      2930      2940
                 =========+=========+=========+=========+=========+=========+
Ec-14_006860     HVSFTMEGQISSYPEGSDARTNFLWAFLLDLARALGVATSRFTITDMRAGSVVVDFTIEP
MSTRG.54525.1    ------------------------------------------------------------
                                                                             


                       2950      2960      2970      2980      2990      3000
                 =========+=========+=========+=========+=========+=========+
Ec-14_006860     TGSLADQSVTELLTNLDEQISDESSSLRNGNVTSAAKGLTYTTVVEADTTPTDSSAAVIG
MSTRG.54525.1    ------------------------------------------------------------
                                                                             


                       3010      3020      3030      3040      3050      3060
                 =========+=========+=========+=========+=========+=========+
Ec-14_006860     LSVVEYRPKGLFGFTSTVWYTTEASGTVTLTIARDHGTKGVMDIGYTTADGSATGGDDYT
MSTRG.54525.1    ---------------SVLWYTTEASGMVTLTIERDHGVKGVMDIGYSTSDGSATGGDDYV
                                #############################################


                       3070      3080      3090      3100      3110      3120
                 =========+=========+=========+=========+=========+=========+
Ec-14_006860     ALAGTVRFYDGDTSKTVDVPLVDDVETEAHFESFSIALSLEGPINEGAALKATATEAEVL
MSTRG.54525.1    SVASSVRFYEGDISKSVDIQLINDDEMEVHFEFFTVVLSLEGPINVGAALKTTASQATVF
                 ############################################################


                       3130      3140      3150      3160      3170      3180
                 =========+=========+=========+=========+=========+=========+
Ec-14_006860     LYDYGDGVVLADATFSANPKS---SSGGAEDLALGWKITDNGGQSGWVDSIGFAAKDAIF
MSTRG.54525.1    LYDYGDGVVLADARFALQQDSSIAGAGGAEDLALGWTIADNGGHSGWVDSNGFAAKDAVF
                 #####################     ##################################


                       3190      3200      3210      3220      3230      3240
                 =========+=========+=========+=========+=========+=========+
Ec-14_006860     GADEYDDRCDYAATAPCDHDCIFGGGLAELFPATGESPSVIRLNSTGYVTTVDPVSNFPS
MSTRG.54525.1    GADEYDERCDYAATAPCDHDCTFGGGLAELFPTTGRTPSVIRLNSTGSVSTVRAVADFPS
                 ############################################################


                       3250      3260      3270      3280      3290      3300
                 =========+=========+=========+=========+=========+=========+
Ec-14_006860     TAFSLSFWIRGISTSGGRGGGTVVSYVGPNSSPSDFEVLLHNLNGLNLLVHGKFVSAADR
MSTRG.54525.1    DVFSLAFWIRAISASGGRGGGTVVSYAATDASPNDYEILLHNLKGLSLIVHGKFVSATDR
                 ############################################################


                       3310      3320      3330      3340      3350      3360
                 =========+=========+=========+=========+=========+=========+
Ec-14_006860     YDGPSGGDLGGIRTGIDVARDGAWHHVTVAWRSADGRVDAFYDGARVFDGGPYKAGMTLS
MSTRG.54525.1    YDGSSGGDLGGIKTGVDASLDEAWHHVAIAWRSADGRVDAFIDGARVFEGGPYKTGAVLS
                 ############################################################


                       3370      3380      3390      3400      3410      3420
                 =========+=========+=========+=========+=========+=========+
Ec-14_006860     TGGRLVLGQAQSSDCTSAQDVVTANVSTSDP-LECEVVSGLKGPGGLEAEVQHLRLWSKF
MSTRG.54525.1    AGGTLVLGQTHSSGCGLTEDTAAAYMSTSDPPPVCGIGNEQSGPGGLEAEVQHLRLWSKF
                 ###############################   ##########################


                       3430      3440      3450      3460      3470      3480
                 =========+=========+=========+=========+=========+=========+
Ec-14_006860     VTADEVAQQMHEPFEGNTVGQVLHWDFTPASMEGRFVEDSSGVGESVSGEANHGLLSENG
MSTRG.54525.1    VTAEEVAQQMQEPFEGNSVGQVLHWDFTPTSMKDRIVEDASGVGASGSGQANDGLLSGSG
                 ############################################################


                       3490      3500      3510      3520      3530      3540
                 =========+=========+=========+=========+=========+=========+
Ec-14_006860     VGLVQASPSLNPGYPCGVVHAGIWRFAGSEEFVTGLRRGTSYGGRLQFRLAYSAASGDIR
MSTRG.54525.1    LDLVQASPSLNPGYPCGVVHSNIWRFAASEEFVSGLRSSMSYGGRLQFRLAYSAAIGDER
                 ############################################################


                       3550      3560      3570      3580      3590      3600
                 =========+=========+=========+=========+=========+=========+
Ec-14_006860     STRGAVVIRSRGGQEISWNLIFDVAEAVPGHWTHVSVVLREDHGWLHEPLGEDVTMAEMK
MSTRG.54525.1    STRGAVIIRSRGGQEISWNLIFNVSGALLGQWTHISVVLREDHGWLVEPLGDDVQMGEMK
                 ############################################################


                       3610      3620      3630
                 =========+=========+=========+=========
Ec-14_006860     EILANASDMLIRGDLRVFGRSGGGQEVVYLQDVRLLAKA
MSTRG.54525.1    GILADASEMLIRGDIRVFGRSGGGQEVVYLQDVQLLAKA
                 #######################################
```

```
Parameters used
Minimum Number Of Sequences For A Conserved Position: 2
Minimum Number Of Sequences For A Flanking Position: 2
Maximum Number Of Contiguous Nonconserved Positions: 8
Minimum Length Of A Block: 10
Allowed Gap Positions: None
Use Similarity Matrices: Yes
```

```
Flank positions of the 3 selected block(s)
Flanks: [3016  3141]  [3147  3391]  [3395  3639]  

New number of positions in Ec-14_006860.aa.fasta.trans.aa_ali.fasta-gb:  616  (16% of the original 3639 positions)
```
